# Absent or diminished pedal pulses and estimated GFR decline in patients with diabetic kidney disease

**DOI:** 10.1080/0886022X.2019.1638273

**Published:** 2019-07-29

**Authors:** Nivetha Subramanian, Jennifer Xu, Laure Sayyed Kassem, Michael Simonson, Niraj Desai

**Affiliations:** a Division of Nephrology and Hypertension, Case Western Reserve University School of Medicine, Cleveland, OH, USA;; b Division of Endocrinology, Case Western Reserve University School of Medicine, Cleveland, OH, USA;; c Department of Medicine, University Hospitals Cleveland Medical Center and Louis Stokes VA Medical Center, Case Western Reserve University School of Medicine, Cleveland, OH, USA

**Keywords:** Peripheral arterial disease, type 2 diabetes, CKD, renal failure

## Abstract

**Background:** Peripheral artery disease (PAD) is a complication of type 2 diabetes that leads to critical limb ischemia and amputation. We tested whether absent or diminished pedal pulses (ADPPs) predicts subsequent renal functional decline in patients with diabetic chronic kidney disease (CKD). We also examined the association between urinary biomarkers and ADPP as well as worsening CKD.

**Methods:** Using a prospective longitudinal design, we studied 91 patients with type 2 diabetes and estimated glomerular filtration rate (eGFR) from 7 to 146 mL/min/1.73 m^2^. Baseline pedal pulses were assessed by standardized history and physical examination. The primary endpoint was decline in eGFR >30%. Potential confounders of the relationship between pedal pulses and eGFR were assessed by multivariable logistic regression.

**Results:** Of 91 participants (median age 58 (range 30–83); median eGFR 72.4 ± 33.4 mL/min/1.73 m^2^), 43% had at least one ADPP. Baseline ADPP associated with increased risk of greater than 30% decline in eGFR (OR= 3.67, *p* = .004). This association remained significant (OR = 3.09, *p* = .029) after adjustment for traditional risk factors of renal function decline in diabetic kidney disease (DKD). In addition, urinary endothelin-1 (ET-1) was higher among patients with ADPP (*p* =.0006) and associated with eGFR decline greater than 30% (adjusted OR = 1.81, *p* = .035).

**Conclusions:** ADPP is a strong predictor of decline in renal function in type 2 diabetes. Patients with type 2 diabetes and abnormal pedal pulses should be screened for DKD and monitored closely for progression of CKD.

## Introduction

Vascular disease reflects a combination of pathophysiologic processes that culminate in altered structure and function of vessels, leading to arterial insufficiency. Traditional risk factors for the development of these diseases include older age, diabetes, hypertension, hyperlipidemia, and smoking are also common in patients with chronic kidney disease (CKD) [1]. Moreover, a growing number of studies have shown a high prevalence of vascular disease in patients with CKD, particularly in those patients with albuminuria [2–7]. The excessive risk of these diseases in CKD is also associated with additional independent risk factors including inflammatory markers, oxidative stress, and sub-optimally controlled diabetes [2]. Chronic kidney disease itself may be a marker for mechanisms beyond shared cardiovascular risk factors that lead to vascular disease.

The extent to which the association between CKD and vascular disease varies in specific etiologies of CKD, such as diabetic kidney disease (DKD), is unknown. It is known, however, that patients with DKD suffer from high rates of critical limb ischemia and amputation [3]. Thus, the need for screening in this patient population cannot be understated.

Several studies have suggested that peripheral arterial disease (PAD) and arterial stiffness are associated with worsening progression of renal disease and cardiovascular disease [4,5]. The detection of PAD is often reliant on diagnostic maneuvers that may have limited accuracy in the diabetic patient, require additional appointments and time within the healthcare system, require the use of nephrotoxic contrast agents, and may not be widely or routinely available to all. Early and rapid detection of vascular disease clinically using a focused, standardized history and physical exam may allow for more targeted and efficient screening in under-recognized, yet high risk groups, such those with DKD, in order to maximize the potential of preventive pharmacologic therapy and minimize the risk of future complications arising from vascular disease [6,7]. Previous studies support the notion that simple historical factors such as patient age and self-reported claudication along with physical examination of peripheral lower extremity pulses and venous filling time reliably predict the presence of vascular disease in diabetics even without the use of ABI [8].

Urinary biomarkers have been extensively studied in AKI and may reflect early cardiovascular disease in CKD patients. For instance, lipocalin-2 also known as neutrophil gelatinase-associated lipocalin (LCN2NGAL as listed in this study) is expressed by neutrophils. One of the earliest kidney markers of ischemic and nephrotoxic injury, it has been studied as a marker of CKD progression [9]. Urinary angiotensinogen (AGT) levels reflect the activity of the renin angiotensin system (RAS) which is thought to mediate tissue injury in patients with CKD [10]. MCP-1 is detectable in the urine of patients with various renal diseases and may reflect underlying inflammatory damage. Urinary transforming growth factor-beta (TGF-β) is a multifunctional polypeptide that is known to be involved in renal sclerosis and the pathogenesis of diabetic nephropathy [11,12]. ET-1 is a potent vasoconstrictor and has been found to be involved in both CKD and CVD via endothelial dysfunction as well as through oxidative stress and inflammation [13]. Growth-differentiation factor-15 (GDF-15) is related to the TGF-B cytokine family and is expressed during inflammation and tissue injury. Several studies have examined its use as a prognostic marker in cardiovascular disease including heart failure [14,15]. Furthermore, in mice models, GDF-15 has been thought to regulate damage in renal tissue [16]. In light of the high prevalence of vascular disease in patients with CKD and the potential for shared mechanisms of injury, it is possible that these urinary biomarkers may be associated with ADPP and further estimated glomerular filtration rate (eGFR) decline.

There is a dearth of studies that have examined the association between urinary biomarkers, peripheral vascular disease, and further renal function decline. Hence, we hypothesized that absent or diminished peripheral pulses (ADPPs) predict eGFR decline in a cohort of patients with DKD. In addition, we examined whether the aforementioned urinary biomarkers may associate with ADPP and further eGFR decline.

## Methods

### Study sample and design

**Figure 1.**
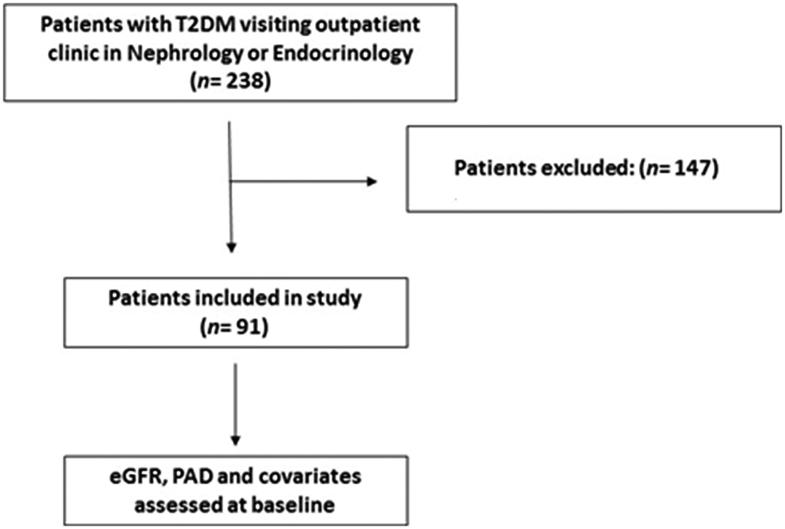
Flow diagram for participant enrollment and cross-sectional assessment of PAD, eGFR, and all other covariates.

Ninety-one participants with type 2 diabetes were recruited from a consecutive sample of 238 patients in outpatient clinics in endocrinology and nephrology at University Hospitals Cleveland Medical Center. Studies were approved by the Institutional Review Board at University Hospitals Cleveland Medical Center, and all participants provided written informed consent. Inclusion criteria were: eGFR > 7 mL/min/1.73 m^2^ as estimated by the CKD-EPI equation [17], age 21–85 years; diagnosis of diabetes using the revised criteria of the American Diabetes Association [18] or use of insulin or oral hyperglycemic agents. Exclusion criteria were a concurrent diagnosis of non-DKD, unwillingness or inability to provide informed consent, concurrent use of dialysis, pregnancy, lactation, substance abuse, fever, systemic and urinary-tract infections, or inflammatory disease (Figure 1).

### Vascular disease assessment

Standardized history and physical examination were used to assess peripheral pulses [7,8,19]. Briefly, examiners graded palpable dorsalis pedis (DP) and posterior tibialis (PT) pulses as absent, diminished, or normal. Barely palpable pulses were coded as diminished. Pulses in each lower limb were coded as absent only if both the DP and PT were absent in the same foot, and as diminished if both pulses were diminished or if either pedal pulse was diminished and the other was absent [8]. Participants were classified as ADPP if at least one pulse in one foot was absent or diminished. Peripheral vascular disease was excluded if peripheral pulses were normal in both lower limbs and there were no femoral bruits. When using ankle-brachial index (ABI) <0.90 as a gold standard, this physical exam-based assessment identifies peripheral vascular disease with negative predictive values of 85–93% and positive predictive values of 80–84% [7,8,19].

Additional assessment of signs and symptoms associated with diminished limb perfusion were conducted using capillary refill time and the Edinburgh Claudication Questionnaire [20]. To determine capillary refill time, the examiner applied firm pressure to the plantar aspect of the great toe for 5 s. After release, time for normal skin color to return was assessed and measurements exceeding 5 s were coded as delayed refill [8].

### Kidney measures and outcomes

Glomerular filtration rate was estimated from serum creatinine using the CKD-EPI equation (eGFR). Albumin excretion was measured as the ratio of urine albumin/urine creatinine in a spot urine specimen. The primary outcome of interest was eGFR decline greater than 30% expressed as a categorical variable [21]. An eGFR decline greater than 30% was used due to its utility in identifying patients at increased risk for all-cause mortality and end-stage renal disease [21]. Percentage change in estimated GFR was calculated as: (last estimated GFR − first estimated GFR)/(first estimated GFR)×100% [22]. All patients had at least two follow-up visits with measurement of eGFR. Participants were followed for an average of 4.8 ± 1.4 years after the initial screening visit, and none were lost to follow-up.

### Urinary biomarkers

Urinary biomarkers were collected at an initial screening visit by trained personnel (∼40 mL midstream). Aliquots of the urine were sent to University Hospitals Case Medical Center central laboratory for urine albumin and creatinine measurements. The remaining samples (∼30 mL) were processed as described by the Human Kidney and Urine Proteome Project and the European Urine and Kidney Proteomics Initiative [23]. Conventional urine dipstick analysis was performed (Multistix 8 SG, Bayer, Tarrytown, NY) after which the specimen was transferred to a 50 mL conical tube and centrifuged at 1500×*g* for 10 min. Smaller aliquots (10 mL) were then centrifuged at 10,000×*g* to remove particulates; these were then aliquoted and stored at −80 °C. Before further analysis, urine pH was adjusted to 8.0 with 1 M Tris buffer 9, pH 8.0 to help solubilize aggregates that may form after thawing. Processed samples were frozen within 3 h of collection. Urine biomarkers (LCN2NGAL, AGT, TGF-β, ET-1, MCP-1, GDF-15) were measured by ELISA (R&D Systems, Minneapolis, MN) and normalized for urine creatinine as reported previously [24]. Assays were performed within 8 months of urine collection and after no more than one freeze–thaw cycle.

### Covariate assessment

In our analyses, we evaluated the association between ADPP and eGFR decline of greater than 30% from baseline, considering covariates traditionally associated with eGFR decline such as baseline eGFR, duration of diabetes, age, hypertension, urine ACR, and glycemic control.

### Statistical analysis

Continuous variables are presented as median ± standard deviation, and categorical variables as proportions. Candidate predictors of vascular disease were selected based on a literature review, *a priori* consensus of clinical importance and prevalence in the study population. The relationship between predictor variables and the pedal pulses was assessed using bivariate logistic regression (odds ratio (OR) and 95% CI). All continuous variables were assessed for co-linearity. Adjustment for potential confounders was performed in Stata (v 13) by multivariable logistic regression.

## Results

### Participant selection and baseline characteristics

Among the 91 patients, the median age was 58 (range 30–83), median eGFR was 70.6 ± 30.5 mL/min/1.73 m^2^, and 43% had ADPP as determined by standardized physical examination. Age, female sex, black race, and body mass index were similar in both ADPP and normal pedal pulse cohorts (*p=* .311, .416, .808, and .993, respectively). Duration of diabetes and HbA1c was similar in patients with and without ADPP (*p* = .740 and .189, respectively). Patients with ADPP had modestly lower baseline eGFR (*p* = .003) but similar urine albumin/creatinine ratio (*p* = .345). Hypertension, defined as systolic BP > 140 or BP > 140/90 mm Hg on antihypertensive medications, was more common in patients with ADPP (*p* = .031). These findings are summarized in Table 1.

**Table 1.** Patient characteristics, total and stratified by pedal pulses.

		Pedal pulse status		
	All	Normal	ADPP	*p* Value
*N*	91	52	39	–
Age (years)	58 ± 11	56 ± 11	59 ± 11	.311
Female (%)	49 (54)	26 (50)	23 (59)	.416
Black (%)	52 (57)	29 (56)	23 (59)	.808
Duration diabetes (years)	7.0 ± 7.3	7.0 ± 7.2	7.0 ± 7.5	.740
HbA1c (%)	7.8 ± 1.8	7.6 ± 1.8	7.9 ± 1.7	.189
Insulin (%)	45 (49)	25 (49)	20 (51)	.256
Oral anti-glycemics (%)	46 (51)	23 (44)	23 (59)	.139
BMI (kg/m^2^)	30.0 ± 6.8	30.0 ± 7.2	29.0 ± 6.4	.993
Baseline eGFR (mL/min/1.73 m^2^)	70.6 ± 30.538	80.8 ± 29.7	62.3 ± 28.8	.003
Smoking (%)		32	43	.368
Urine ACR (mg/g)	75 ± 521	47 ± 338	77 ± 683	.345
Systolic BP (mm Hg)	138 ± 15	138 ± 14	140 ± 16	.231
Hypertension (%)	79 (87)	42 (80)	37 (95)	.031
Dyslipidemia (%)	52 (60)	26 (50)	26 (67)	.205
CAD (%)	26 (29)	15 (28)	11 (29)	.945
RAS Inhibitors (%)	62 (68)	33 (64)	29 (74)	.330

Data are reported as *n* (%), mean ± SD or median, interquartile range. *p* Values are comparing with and without PAD estimated by the Wilcoxon–Mann–Whitney test or Chi-Square as appropriate.

### Baseline pedal pulses and renal function decline

ADPP was associated with an increased risk of eGFR decline greater than 30% (unadjusted OR = 3.67, *p* = .004). After adjustment for traditional risk factors associated with eGFR decline including baseline eGFR, age, duration of diabetes, glycemic control, and albuminuria, ADPP was still a strong predictor of eGFR decline greater than 30% (adjusted OR = 3.09, *p* =.029). Furthermore, ADPP was not associated with albuminuria measured using the albumin/creatinine ratio (*p* = .428). These results are summarized in Table 2.

**Table 2.** Unadjusted and adjusted odds ratios for development of eGFR decline >30%.

Predictor	Unadjusted			Adjusted^a^		
	Odds ratio	95% CI	*p*	Odds ratio	95% CI	*p*
ADPP	3.67	1.50–8.96	.004	3.09	1.12–8.52	.029
Baseline eGFR	0.99	0.98–1.00	.159	0.98	0.96–1.00	.127
Age	1.0	0.96–1.04	.920	0.96	0.91–1.02	.151
Diabetes duration, years	1.04	0.98–1.10	.217	1.08	1.00–1.16	.050
Urine ACR	1.0	1.0–1.0	.342	1.00	1.00–1.00	.117
HbA1C	1.02	0.80–1.30	.864	1.06	0.77–1.46	.714
HTN	8.07	0.99–65.60	.051	6.35	0.60–66.7	.124

Odds ratios, 95% CIs and *p* values for predictors of PAD in the cross-sectional analysis. The Hosmer–Lemeshow goodness-of-fit for the adjusted model, 6.50, *p*= .591.

a Adjusted for other variables listed in table. For instance, in the adjusted column for HbA1C, the association between HbA1C and eGFR decline greater than 30% after adjustment for PAD, age, duration of diabetes, urine ACR, and hypertension.

### Urinary biomarkers and renal function decline

As shown in Table 3, endothelin-1 (ET-1) was higher among patients with ADPP (*p* =.0006). In addition, as shown in Table 4, ET-1 was associated with eGFR decline greater than 30% and this association remained significant after controlling for independent risk factors associated with eGFR decline such as baseline eGFR, duration of diabetes, microalbuminuria, age, hypertension (adjusted OR = 1.81, *p* =.035).

**Table 3.** Urinary biomarkers, total and stratified by PAD.

		Pedal pulse status		
	All	Normal pulses	ADPP	*p* Value
ET-1	3.5 ± 1.9	2.9 ± 2.0	4.5 ± 1.6	.0006
AGT	21.4 ± 29.0	18.5 ± 30.0	26.6 ± 28.3	.603
TGF-B	44.5 ± 49.2	35.7 ± 59.7	66.4 ± 29.9	.183
MCP1	127.2 ± 69.8	130.6 ± 70.0	123 ± 69.6	.529
GDF15	14.2 ± 12.3	10.6 ± 11.9	21.6 ± 12.4	.056
LCN2NGAL	39.5 ± 37.6	39.3 ± 43.6	41.1 ± 28.4	.941

Data are reported as *n* (%), mean ± SD or median, interquartile range. *p* Values are comparing with and without pedal pulses estimated by the Wilcoxon–Mann–Whitney test or Chi-Square as appropriate.

**Table 4.** Unadjusted and adjusted odds ratios for development of eGFR decline >30% with elevated baseline ET-1.

Predictor	Unadjusted			Adjusted		
	Odds ratio	95% CI	*p*	Odds ratio	95% CI	*p*
ET-1	2.05	1.43–2.95	.000	1.81	1.04–3.14	.035
Baseline eGFR	0.99	0.98–1.00	.159	1.02	0.97–1.07	.442
Diabetes duration, years	1.04	0.98–1.10	.217	1.12	0.97–1.29	.110
Urine ACR	1.00	1.00–1.00	.342	1.00	1.00–1.00	.217
HbA1C	1.02	0.80–1.30	.864	1.66	0.8–3.23	.132
HTN	8.07	0.99–65.6	.051	9.55	0.28–329	.212
Dyslipidemia	0.72	0.25–2.09	.551	0.42	0.07–2.56	.345

## Discussion

We studied the relationship between pedal pulses and eGFR decline greater than 30% in a cohort of DKD patients. Our study showed that ADPP is associated with eGFR decline greater than 30% (OR = 3.67, *p* = .004). This relationship persisted (OR = 3.09, *p* =.023), after adjustment for traditional risk factors for eGFR decline such as age, duration of diabetes, urine ACR, glycemic control, hypertension, and baseline eGFR.

The association between PAD and eGFR has been extensively studied. Baber et al. found that participants with a reduced eGFR (<60 mL/min/1.73 m^2^) had a significantly higher prevalence of PAD (14.8%) compared with those with eGFR > 60 mL/min/1.73 m^2^ (3.6%) [25]. Similarly, in a study involving 955 hypertensive subjects, nearly two-thirds of whom were diabetic, Mostaza et al. showed that patients with eGFR < 60 mL/min/1.73 m^2^, had a significantly higher prevalence of PAD as determined by ABI [26].

Some studies have investigated PAD and its association as a predictor of renal function decline. One study by Foster et al. found that low ABI was associated with the development of eGFR decline as well as incident CKD [27]. In their study of 897 atrial fibrillation patients, Violi et al. found that ABI ≤ 0.9 was independently associated with a rapid decline in renal function as well as incident CKD [28].

Many previous studies have used the ABI, a noninvasive test that describes the ratio between systolic blood pressure in the lower and upper extremities to examine PAD. The test is limited in its sensitivity to detect PAD in the diabetic patient, particularly in the presence of medial arterial calcification, a condition that causes arterial stiffness. Even in the presence of significant PAD, ABI values in such patients may be elevated to ‘falsely normal’ or even abnormally high values, resulting in undetected PAD in many diabetic patients [29]. In addition to these reasons, we used a standardized history and physical examination due to its simplicity, zero cost and patient centered appeal of avoiding additional appointments and time within the healthcare system. In a study of 11,120 patients with type 2 diabetes, Mohammedi et al. found that even a single absent pedal pulse significantly increased the patient’s risk of cardiovascular disease as well as progression to renal disease [30]. There are some limitations to palpation of pedal pulses including anatomic variability and user-dependent variability in assessing pulses [31]. Despite these limitations, absent pedal pulses can reflect early manifestations of vascular disease.

Our findings suggest that pedal pulses could be used as a simple marker to identify DKD patients at increased risk of eGFR decline. We postulate two potential mechanisms by which absent or diminished pedal pulses (ADPPs) may reflect eGFR decline. First, abnormal pedal pulses may reflect generalized cardiovascular disease and atherosclerosis. It is possible that the widespread atherosclerosis will also involve the renal vasculature, leading to ischemic renal disease. In a study involving a sample of US Medicare patients, individuals with peripheral vascular disease had 3.96 greater odds for atherosclerotic renovascular disease, after adjustment for age, gender, race, and comorbid conditions [32]. Thus, abnormal pedal pulses may reflect a heavy burden of atherosclerosis that coincides with a greater tendency toward declining renal function.

Another potential mechanism involves shared risk factors between DKD and cardiovascular disease that lead to preexisting systemic disease, and this underlying systemic disease may contribute independently to the development of both vascular disease and renal function decline. Previous studies have identified the role of the neurohumoral pathway in CKD, CHF, and T2DM [33]. We postulate that urinary biomarkers, particularly urinary ET-1, possibly illuminate this mechanism. In Tables 3 and 4, we demonstrated that the ADPP group had higher levels of ET-1, and ET-1 was associated with eGFR decline greater than 30%. A potent vasoconstrictor, ET-1 is released from both endothelial and renal tubular cells and is responsible for proliferation, fibrosis, and inflammation [34,35]. Several studies have suggested that patients with diabetes and CKD have higher levels of ET-1, reflecting coincidental endothelial and renal dysfunction that can lead to vascular remodeling and ultimately, ischemic nephropathy and PAD [36–43]. In a rodent study, kidney ET-1 was found to mediate the development of renal damage, while a separate study found that urinary ET-1 was a marker of severity of renal damage [44,45]. It is possible that baseline urinary ET-1 may reflect underlying inflammation and vascular endothelial dysfunction that leads to the development of both vascular disease and eGFR decline. These findings add to a growing body of data that identify pedal pulses as a novel predictor for renal function decline among DKD patients.

There are several limitations to our study. Our results may be restricted by the relatively small sample size of 91 patients. Furthermore, renal biopsies were not performed in the patient cohort. As a result, the diagnosis of DKD was subject to interpretation of historical and laboratory data. The diagnosis of ADPPs, an indicator of vascular disease, by standardized history and physical examination does contain some subjective elements. Patients with known severe peripheral vascular disease were not excluded from the study. Furthermore, we did not analyze the renal function decline to look for episodes of AKI. Future directions for this project include exploring the pathophysiologic mechanisms, such as inflammatory markers and oxidative stress, underlying the complex interplay between DKD and vascular health. Furthermore, we would like to identify if urinary biomarkers can be utilized to identify patients at greatest risk for vascular disease and renal function decline.

## Conclusions

In summary, our findings highlight and support the use of ADPP to potentially identify patients at risk for development of further renal functional decline. In addition, our study supports the notion that urinary biomarkers, particularly ET-1, may reflect the underlying mechanism by which ADPP may lead to further renal decline. Targeted screening resulting in timely diagnosis of vascular disease and ultimately, potential modification of risk factors may help those patients with DKD who are at heightened risk for further renal function decline.
